# Baseline immune signature score of Tregs × HLA-DR^+^CD4^+^ T cells × PD1^+^CD8^+^ T cells predicts outcome to immunotherapy in cancer patients

**DOI:** 10.3389/fimmu.2022.1054161

**Published:** 2022-11-28

**Authors:** Rebekka Mispelbaum, Sandra Tessa Hattenhauer, Stefanie Andrea Erika Held, Peter Brossart, Annkristin Heine

**Affiliations:** Department of Oncology, Hematology, Immune-Oncology and Rheumatology, University Hospital Bonn, Bonn, Germany

**Keywords:** prediction, response, immunotherapy, peripheral immune cells, cancer, immune signature

## Abstract

**Background:**

The use of immunotherapy (IT) is rapidly increasing across different tumor entities. PD-L1 expression is primarily used for therapy evaluation. The disadvantages of PD-L1 status are spatial and temporal heterogeneity as well as tumor type-dependent variation of predictive value. To optimize patient selection for IT, new prediction markers for therapy success are needed. Based on the systemic efficacy of IT, we dissected the immune signature of peripheral blood as an easily accessible predictive biomarker for therapeutic success.

**Methods:**

We conducted a retrospective clinical study of 62 cancer patients treated with IT. We assessed peripheral immune cell counts before the start of IT *via* flow cytometry. The predictive value for therapy response of developed immune signature scores was tested by ROC curve analyses and scores were correlated with time to progression (TTP).

**Results:**

High score values of “Tregs ÷ (CD4^+^/CD8^+^ ratio)” (Score A) and high score values of “Tregs × HLA-DR^+^CD4^+^ T cells × PD1^+^CD8^+^ T cells” (Score B) significantly correlated with response at first staging (*p* = 0.001; *p* < 0.001). At the optimal cutoff point, Score A correctly predicted 79.1% and Score B correctly predicted 89.3% of the staging results (sensitivity: 86.2%, 90.0%; specificity: 64.3%, 87.5%). A high Score A and Score B statistically correlated with prolonged median TTP (6.13 *vs*. 2.17 months, *p* = 0.025; 6.43 *vs*. 1.83 months, *p* = 0.016). Cox regression analyses for TTP showed a risk reduction of 55.7% (HR = 0.44, *p* = 0.029) for Score A and an adjusted risk reduction of 73.2% (HR = 0.27, *p* = 0.016) for Score B.

**Conclusion:**

The two identified immune signature scores showed high predictive value for therapy response as well as for prolonged TTP in a pan-cancer patient population. Our scores are easy to determine by using peripheral blood and flow cytometry, apply to different cancer entities, and allow an outcome prediction before the start of IT.

## Introduction

The use of checkpoint inhibitors as monotherapy as well as concomitant to chemotherapy is rapidly increasing across different tumor entities (1). Tumor cells suppress antitumor immunity *via* different signaling pathways including programmed death-ligand 1 (PD-L1) and programmed cell death protein 1 (PD-1) (2). By blocking these molecules, immunotherapy (IT) leads to an enhancement of CD8^+^ T cell activity resulting in antitumor immunity (2, 3). Local antitumor immune response depends on an interaction with and contribution of the systemic immune system. Peripheral immune cells augment, sustain, and reactivate local IT effects by interaction with the tumor microenvironment. For example, circulating CD8^+^ T cells are assumed to migrate into the tumor microenvironment enhancing local antitumor immunity (4, 5).

Several FDA approvals of IT are based on PD-L1 expression for patient selection. However, the predictive value of PD-L1 status varies widely depending on the tumor type (6). Further disadvantages of PD-L1 status are spatial and temporal heterogeneity, lack of standardized laboratory methods, and different PD-L1 staining cutoffs in trials (7).

New prediction markers are urgently needed to optimize selection of patients profiting from IT and to avoid severe adverse events in non-responders. Beyond PD-L1 status, research has focused on the composition of tumor-infiltrating immune cells showing relevance for therapy response (8). However, this method could not be established in everyday clinical practice. In general, tumor tissue-based analysis is limited by feasibility of re-biopsies with corresponding risk.

Due to the crucial role of systemic antitumor immunity for effective tumor control, there is an increasing interest in the immune signature of the peripheral blood as a predictive biomarker for therapeutic success for clinical routine (5). Previous studies have investigated immune cell lines or laboratory parameters of peripheral blood in specific cancer types without testing across different tumor entities, focusing on changes of biomarkers during IT without predictive value before therapy start. Investigated study populations were mainly treated with single IT without additional chemotherapy or radiotherapy, which only partially reflects IT use in clinical practice (8–10).

The objective of this study was to establish immune signature scores of peripheral blood cells predicting success of IT before therapy start in pan-cancer population.

## Materials and methods

We conducted a retrospective clinical study of patients treated with IT for metastatic cancer at a single tertiary care center between May 2015 and October 2021. Inclusion criteria were at least one radiological staging after start of IT and one flow cytometry testing. Patients with different tumor entities were enrolled, mainly with lung cancer, head and neck cancer, and skin cancer.

IT could be applied as monotherapy or IT doublet as well as concomitant to radiotherapy or chemotherapy. Investigated drugs were the PD-L1 inhibitor atezolizumab, the PD-1 inhibitors nivolumab and pembrolizumab, and the cytotoxic T-lymphocyte-associated protein 4 (CTLA-4) inhibitor ipilimumab.

Initial therapy response was evaluated by the first conducted CT or MRI scan after treatment start (median time: 63 days after first IT application) according to the local hospital guidelines. Therapy response was defined as stable disease, partial response, or complete response. Time to progression (TTP) was calculated from the date of start of IT to documented progress and censored at the last visit until which no disease progression was observed. The follow-up time was limited to 24 months and stopped in case of documented tumor progression or death.

For each cancer patient, we assessed a detailed manual chart review. Levels of serologic parameters and immune cell subsets (tested by flow cytometry) were analyzed. The number of patients varied for the observed parameters, depending on the type of laboratory tests performed upon treatment start. The median time of flow cytometric analysis was 22 days before start of IT.

### Flow cytometry analysis

For flow cytometry, blood was collected in heparin tubes and processed within 24 h by a flow cytometer (model: Navios EX; Beckman Coulter, Krefeld). Incubation of 100 µl of whole blood was performed with dried custom-designed format reagents (DuraClone tubes; Beckman Coulter, Krefeld). According to the manufacturer’s protocol, lyse and fix solution was applied. The antibodies used were CD3-AA700, CD4-APC, CD8-KrOr, CD14-AA750, CD16-FITC, CD25-PE, CD56-ECD, CD127-PC7, HLA-DR-PB, and PD1-PC5.5. From August 2017 to September 2018, the antibodies used were CD3-FITC, CD4-PC7, CD14-PE, CD16-PC5, CD25-PC5, CD127-PE, and HLA-DR-FITC. CD4^+^ and CD8^+^ subsets were gated from the lymphocyte gate based on CD3 and CD4 expression for CD4^+^ T cells and CD3 and CD8 expression for CD8^+^ T cells. The lymphocyte gate was identified by forward and sideward scatter. HLA-DR^+^CD4^+^ T cells and PDL1^+^CD8^+^ T cells were gated from CD3^+^ lymphocytes based on HLA-DR^+^ and CD4^+^ expression and PD1^+^ and CD8^+^ expression, respectively. Tregs were defined as CD3^+^CD4^+^CD25^+^CD127^-^ cells gated from the lymphocyte gate.

### Statistical analysis

To evaluate the association between treatment response and laboratory parameters, analyses by Mann–Whitney *U* test, Student’s *t*-test, and Kruskal–Wallis test were applied. In case of a statistical and expected pathophysiological relationship, the variables were combined in predictive scores. To measure the predictive power of the individual score, receiver operating characteristic (ROC) curves were generated. The optimal cutoff point of the scores was defined as the point at which the Youden index was maximized by the ROC curve and was calculated by the formula “J = sensitivity + specificity − 1”. Comparison of area under the curve (AUC) values for ROC curves was calculated by DeLong test.

TTP curves were generated by the non-parametric Kaplan–Meier method and compared with log-rank test. Correlations were tested by simple Cox regression analyses. In case of statistically and clinically significant relationship, variables were included in multiple Cox regression to analyze the robustness of their prognostic values for TTP after adjustment for covariates.

All analyses and figures were performed using STATA software (version 15.1). A *p*-value < 0.05 was considered as statistically significant. The study was conducted in accordance with the Declaration of Helsinki and was approved by the ethics committee of the Medical Department of the University of Bonn (#340/21). Only previously documented data and routine diagnostic interventions were analyzed; no informed consent was needed. All patients were anonymized through the use of codes.

## Results

### Baseline characteristics

A total of 62 patients treated with IT for metastatic cancer were analyzed. The mean age was 63 years (range: 34–90 years); 71.0% of patients were men and 29.0% were women; 59.7% of patients had a documented ECOG performance status 0–1 before the start of IT. The main documented tumor types were lung cancer (24.2% NSCLC, 8.1% SCLC), head and neck cancer (19.4%), skin cancer (6.5% melanoma, 6.5% non-melanoma skin cancer), and urinary tract cancer (9.7%); 25.8% of patients were treated with chemotherapy in addition to IT, and 21.0% of patients were treated with radiotherapy; 24.2% of patients received IT as first-line treatment and 25.8% received IT as second-line treatment. Initial therapy response was evaluated by the first conducted CT or MRI scan after treatment start, with a median time of 63 days after the first IT application. An initial therapy response was detected in 39 of 62 patients (62.9%), 18 with stable disease (29.0%), 20 with partial response (32.3%), and 1 with complete response (1.6%). 23 patients showed no response to IT (37.1%) (**Table 1**).

**Table 1:** Patient characteristics.

	Total
	(*N* = 62), *n* (%)
**Age**	
Mean [years]	63
Range [years]	34–90
Elderly (>70)	17 (27.4)
**Sex**	
Male	44 (71.0)
Female	18 (29.0)
**ECOG**	
0–1	37 (59.7)
≥2	21 (33.9)
**Tumor type**	
Lung	20 (32.3)
Head neck	12 (19.4)
Skin	8 (12.9)
Urinary tract	6 (9.7)
Breast	3 (4.8)
Others	13 (21.0)
**One-drug immunotherapy**	
Pembrolizumab/nivolumab	41 (66.1)
Atezolizumab	9 (14.5)
Ipilimumab	1 (1.7)
**Two-drug immunotherapy**	
Nivolumab + ipilimumab	11 (17.7)
**Additional therapy**	
Additional chemotherapy	16 (25.8)
Additional radiotherapy	13 (21.0)
**Line of therapy**	
1	15 (24.2)
2	16 (25.8)
3	14 (22.6)
4	7 (11.3)
≥5	10 (16.1)
Mean	2.8
**Number of organs with metastasis**	
Mean	1.8

### Single-cell lines and immune signature scores correlate with response to IT at first staging

A statistically significant correlation with therapy response was seen for higher levels of HLA-DR^+^CD4^+^ T cells (*p* = 0.001), PD1^+^CD8^+^ T cells (*p* = 0.028), PD1^+^NK cells (*p* = 0.001), and Tregs (*p* = 0.049). In patients with a lower CD4^+^/CD8^+^ ratio, we detected a clinically relevant trend of higher response rates (*p* = 0.089). To improve the predictive value for therapy response to IT, we were able to identify two scores based on the previous analyses.

**Figure 1:**
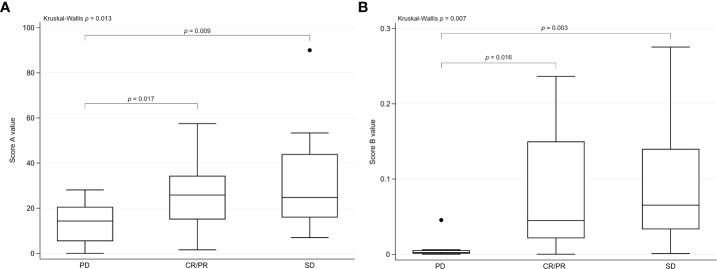
Scores A and B in correlation with response. **(A)** Score A values are presented as box plots with median (line) in relation to response to IT (CR/PR, SD, and PD) at first staging. A statistically significant difference for Score A was detected in patients with CR/PR *vs*. PD (*p* = 0.017) as well as in patients with SD *vs*. PD (*p* = 0.009). In patients with PD, lower score values were detected. **(B)** Score B values are presented as box plots with median (line) in relation to response to IT (CR/PR, SD, and PD) at first staging. A statistically significant difference for Score B was detected in patients with CR/PR *vs*. PD (*p* = 0.016) as well as in patients with SD *vs*. PD (*p* = 0.003). In patients with PD, lower score values were detected. Score A , Tregs ÷ (CD4^+^/CD8^+^ ratio); Score B , Tregs × HLA-DR^+^CD4^+^ T cells × PD1^+^CD8^+^ T cells; IT , immunotherapy; CR , complete response; PR , partial response; SD , stable disease; PD , progressive disease.

Score A was calculated by the division of Tregs by the CD4^+^/CD8^+^ ratio, significantly correlating with response at first staging (*p* = 0.001). To further optimize the precision of Score A, we performed a subclassification of CD4^+^ and CD8^+^ T cells. Score B was calculated by multiplication of Tregs, HLA-DR^+^CD4^+^ T cells, and PD1^+^CD8^+^ T cells. Thereby, Score B showed the strongest significant correlation with response at first staging (*p* < 0.001). In comparison, other developed scores including Tregs, CD4^+^ subsets, and CD8^+^ subsets showed lower statistical significance for prediction of response (**Table 2**). For other tested laboratory parameters and previously published prediction scores, we observed trends but no statistical significance (**Table 2**). For Scores A and B, we observed significantly higher score values in patients with response upon IT compared to patients with progress (**Figure 1**).

**Table 2:** Immune cells, other laboratory markers, and scores in correlation with response to IT.

	Baseline score of responder^a^	Responder *vs*. non-responder*p*-value^b^	Patient (*N*)
**Immune cells**			
Lymphocytes [% of leukocytes]	+	0.132	62
Eosinophiles [% of leucocytes]	+	0.646	61
CD14^+^CD16^-^ monocytes [% of total]	–	0.481	44
CD4^+^ T cells [% of lymphocytes]	–	0.235	52
HLA-DR^+^CD4^+^ T cells [% of total]	+	0.001	28
PD1^+^CD4^+^ T cells [% of total]	+	0.063	28
CD8^+^ T cells [% of lymphocytes]	+	0.154	52
HLA-DR^+^CD8^+^ T cells [% of total]	+	0.146	28
PD1^+^CD8^+^ T cells [% of total]	+	0.028	28
CD4^+^/CD8^+^ ratio	–	0.089	52
Tregs [% of total]	+	0.049*	45
B-cells [% of lymphocytes]	+	0.777	52
NK cells [% of lymphocytes]	+	0.211	52
PD1^+^NK cells [% of total]	+	0.001	28
**Other blood cells**			
Thrombocytes [G/l]	–	0.358	62
**Serum parameters**			
LDH [U/l]	+	0.491	59
CRP [mg/l]	–	0.083	62
**Scores**			
SII	–	0.324	61
NLR	–	0.406	61
dNLR	–	0.392	61
LIPI	–	0.192	58
Thrombocytes ÷ lymphocytes	–	0.221	62
(Tregs) ÷ (CD4^+^/CD8^+^ ratio)^c^	+	0.001	43
(Tregs) × (HLA-DR^+^CD4^+^ T cells)	+	0.001	28
(Tregs) × (PD1^+^CD8^+^ T cells)	+	0.001	28
(HLA-DR^+^CD4^+^ T cells) × (PD1^+^CD8^+^ T cells)	+	0.001	28
(Tregs) × (HLA-DR^+^CD4^+^ T cells) × (PD1^+^CD8^+^ T cells)^d^	+	<0.001	28

a In comparison to non-responder: + higher value, − lower value.

b Was calculated using t-test/* U test.

c Named Score A.

d Named Score B.

IT , immunotherapy; SII , systemic immune-inflammation index; Lipi , lung immune prognostic index; NLR , neutrophil-to-lymphocyte ratio; dNLR , derived neutrophil-to-lymphocyte ratio; × , multiplication; ÷ , division.

### Score A “Tregs ÷ (CD4^+^/CD8^+^ ratio)” predicts response at first staging

**Figure 2:**
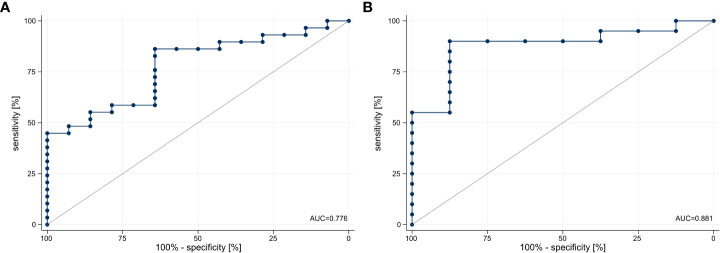
ROC curves for response prediction of Scores A and B **(A)** ROC curve was drawn for response prediction of Score A (AUC = 0.776, 95% CI 0.633–0.919). **(B)** ROC curve was drawn for response prediction of Score B (AUC = 0.881, 95% CI 0.743–1.000). ROC, receiver operating characteristic, Score A = Tregs ÷ (CD4^+^/CD8^+^ ratio), Score B = Tregs × HLA-DR^+^CD4^+^ T cells × PD1^+^CD8^+^ T cells, AUC, area under the curve.

To validate the predictive value of Scores A and B, ROC curves were drawn (**Figure 2**). Score A significantly predicted response at first staging (AUC = 0.776, 95% CI 0.633–0.919, *p* < 0.001). The optimal cut-point value was determined from the ROC curve. Patients with Score A ≥14.78 showed a higher probability for response than patients with Score A <14.78. Sensitivity of Score A was 86.2% and specificity was 64.3%. The positive predictive value was 83.3%, while the negative predictive value was 69.2%. Score A correctly predicted 79.1% of the staging results (**Figure 2A**). The prognostic value of Score A was independent of sex (*p* = 0.259), age (*p* = 0.202), and concomitant chemotherapy (*p* = 0.606).

### Score B “Tregs × HLA-DR^+^CD4^+^ T cells × PD1^+^CD8^+^ T cells” predicts response at first staging

Score B significantly predicted response at first staging (AUC = 0.881, 95% CI 0.743–1.000, *p* < 0.001). Patients with Score B ≥0.01782 showed a higher probability for response than patients with Score B <0.01782. Sensitivity of Score B was 90.0% and specificity was 87.5%. The positive predictive value was 94.7%, while the negative predictive value was 77.8%. Score B correctly predicted 89.3% of the staging results (**Figure 2B**). The prognostic value of Score B was independent of sex (*p* = 0.477), age (*p* = 0.839), and concomitant chemotherapy (*p* = 0.732).

### Scores A and B correlate with time to progression

In our population of 62 patients with a median follow-up of 7.06 months, 48 progression events occurred. The median TTP was 6.03 months.

**Figure 3:**
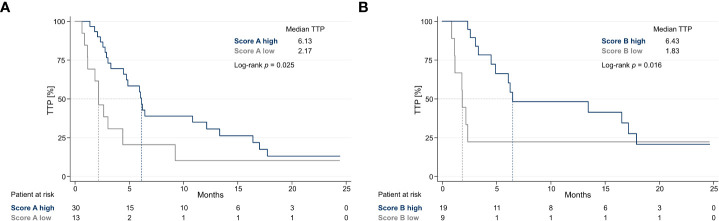
Kaplan–Meier curves of TTP for Scores A and B **(A)** Kaplan–Meier curves show TTP of patients with high (≥14.78) *vs*. low Score A values (<14.78). **(B)** Kaplan–Meier curves show TTP of patients with high (≥0.01782) *vs*. low Score B values (<0.01782). Score A = Tregs ÷ (CD4^+^/CD8^+^ ratio), Score B = Tregs × HLA-DR^+^CD4^+^ T cells × PD1^+^CD8^+^ T cells, TTP, time to progression.

As Kaplan–Meier curves show, higher Score A and Score B values were significantly associated with improved TTP (*p* = 0.025; *p* = 0.016). Median TTP was 6.13 months for patients with a higher Score A and 2.17 months for patients with a lower Score A. Median TTP was 6.43 months for patients with a higher Score B and 1.83 months for patients with a lower Score B (**Figure 3**).

### Higher Score A and Score B values showed a statistically significant risk reduction for TTP

We could show that age (HR = 0.99, *p* = 0.460), sex (HR = 1.58, *p* = 0.139), ECOG (HR = 1.19, *p* = 0.331), type of IT (one drug *vs*. IT doublet; HR = 1.42, *p* = 0.080), concomitant chemotherapy (HR = 0.99, *p* = 0.964), concomitant radiotherapy (HR = 0.58, *p* = 0.165), number of organs with metastasis (HR = 1.17, *p* = 0.184), LDH (HR = 1.00, *p* = 0.247), and CRP at baseline (CRP ≤5 *vs*. >5 mg/l; HR = 1.36, *p* = 0.331) had no statistically significant prognostic value for TTP. Only tumor type (*p* = 0.019) and line of therapy (line of therapy ≤3 *vs*. >3; HR = 2.63, *p* = 0.003), as expected clinical confounders, correlated with TTP. In multiple Cox regression analysis, Score B remained the only independent predictor of superior TTP. Patients with higher Score B values had an adjusted risk reduction of 73.2% for TTP (HR = 0.27, *p* = 0.016). Single Cox regression analysis showed that patients with higher Score A values had a risk reduction of 55.7% for TTP (HR = 0.44, *p* = 0.029), but we could not show statistical significance in multiple Cox regression analysis (*p* = 0.078).

## Discussion

For a large group of different tumor types, IT is a standard treatment used as monotherapy or additional to chemo- or radiotherapy. Despite this increasing use, there is still a lack of biomarkers with predictive value for therapy response (1). Beyond blocking local immunosuppression, IT success is achieved by systemic antitumor immunity, relying on the functionality and composition of the individual immune system (5). Therefore, we developed scores based on the individual immune signature of the patient’s peripheral blood and investigated their predictive value for therapy success of IT. The scores with the best statistical power consisted of three cell lines: CD4^+^ T cells, CD8^+^ T cells, and Tregs. By a precise subclassification of these cell lines, an increase of the predictive power could be achieved.

In detail, we observed a trend of lower CD4^+^/CD8^+^ ratio at baseline (before start of therapy) in IT responders, driven by lower CD4^+^ T cells and higher CD8^+^ T cells. CD8^+^ T cells are considered to be the main effector cells of IT causing direct cytotoxic damage (11). In tumor tissue analyses, high numbers of tumor-infiltrating CD8^+^ T cells correlated with response to IT (12). While total count of peripheral CD8^+^ T cells showed no relevant influence for response, we detected a statistically significant predictive value of the PD1^+^CD8^+^ T cell subpopulation. We assume that the inhibitory receptor PD-1 as an exhaustion marker is expressed on CD8^+^ T cells accessible for IT. Blocking PD-1 by checkpoint inhibitors, the cytotoxic antitumor effect of the CD8^+^ T cells gets unleashed (13). Other studies similarly reported a prognostic value of elevated PD1^+^CD8^+^ T cells in the peripheral blood as a baseline and monitor marker for therapy response in solid tumors (13, 14). Not all subsets of CD8^+^ T cells positively impact IT (12, 15). For example, high senescent CD8^+^ T cells are discussed to negatively impact response to IT (16).

As an inhomogeneous group, CD4^+^ cells may differentiate into immunosuppressive or immune-stimulating cells (17). For the subgroup of HLA-DR^+^CD4^+^ T cells, we could show a statistically significant correlation with therapy response. We assume that in the subsets of HLA-DR^+^CD4^+^ T cells, immune-activating cells outnumber inhibitory cells. The relevance of CD4^+^ T cells for IT success is not completely understood. CD4^+^ T cells may support antitumor immunity by activation of CD8^+^ cells, modulation of the immune system through effector cytokines, and a supposed direct cytotoxic effect (17).

In our study, increased baseline count of Tregs significantly correlated with therapy response at first staging. While high peripheral Treg counts were associated with poorer prognosis in the pre-IT era, an investigation of stromal infiltrating T cells in NSCLC patients showed correlation of increased PD1^+^ Treg counts with response to IT (8, 18). In addition, an elevated Treg count in the peripheral blood was also associated with clinical benefit in NSCLC patients undergoing IT (19). This special observation of Tregs in IT patients is explained by the immune modulatory effects of IT. By deactivating Tregs, IT might reduce tumor-related inhibition of the immune system (2). PD-1 blockade was shown to downregulate intracellular FoxP3 expression of Tregs, indicating an inhibiting effect on this cell population (20). We assume that increased peripheral Tregs indicate a high level of tumor-induced immunosuppression identifying patients susceptible for IT. In line with these findings, nivolumab reduces *in vitro* suppressive capacity of Tregs and additionally enhances CD8^+^ T-cell resistance to Treg suppression (20, 21).

Since the IT-induced antitumor effect is based on a complex interaction of activated and deactivated effector cells, it is mandatory to consider more than one cell line to optimize response prediction. Our developed Score A “Tregs ÷ (CD4^+^/CD8^+^ ratio)” and Score B “Tregs × HLA-DR^+^CD4^+^ T cells × PD1^+^CD8^+^ T cells” showed a high statistically significant correlation with treatment success and correctly classified 79.1% and 89.3% of therapy responses at first staging, respectively. Furthermore, patients with a higher Score A and Score B had a prolonged TTP and a relevant risk reduction for progression of 55.7% and 73.2%, respectively. In multiple Cox regression, Score B remained statistically significant.

The higher predictive value for therapy response and TTP of Score B compared to Score A may be explained by the specific selection of relevant cell subsets for IT success. However, Score A was reliable and might be easier to implement in clinical practice due to its feasibility.

Limitations have to be considered when interpreting our findings. Due to the retrospective small patient cohort, it is necessary to investigate these scores prospectively in a larger patient population. Furthermore, flow cytometry was performed with two different staining protocols over the study period. Limited by the study design, we are not able to make a statement about the correlation between peripheral blood and tumor-infiltrating lymphocytes. Further investigation to understand the high predictive value of the calculated scores is needed, including markers for Tregs like FoxP3 and PD-1.

To our knowledge, these are the first developed immune scores with statistically proven high sensitivity and specificity predicting therapy response as well as TTP in a pan-cancer population treated with IT. In clinical use, these scores might optimize the prediction of therapy success based on the individual immune signature of the patient’s peripheral blood before therapy start.

## Data availability statement

The raw data supporting the conclusions of this article will be made available by the authors, without undue reservation.

## Ethics statement

The studies involving human participants were reviewed and approved by the ethics committee of the Medical Department of the University of Bonn (#340/21). Written informed consent for participation was not required for this study in accordance with the national legislation and the institutional requirements.

## Author contributions

RM: conception and design of the work/acquisition, analysis, and interpretation of data/drafting of the manuscript/statistical analysis. STH: conception and design of the work/acquisition, analysis, and interpretation of data/drafting of the manuscript/statistical analysis. SAH: technical and material support. PB: critical revision of the manuscript. AH: conception and design of the work/analysis, and interpretation of data/critical revision of the manuscript/supervision. All authors contributed to the article and approved the submitted version.

## Funding

Research in the Heine lab is funded by the Deutsche Forschungsgemeinschaft (DFG, German Research Foundation) under Germany’s Excellence Strategy – EXC2151 – 390873048.

## Acknowledgments

We thank Guido Luechters for his statistical support, Jessica von Fischer-Treuenfeld for her help to complete the dataset, Chrystel Flores for her technical support, and Dennis Mispelbaum for his support designing the figures.

## Conflict of interest

The authors declare that the research was conducted in the absence of any commercial or financial relationships that could be construed as a potential conflict of interest.

## Publisher’s note

All claims expressed in this article are solely those of the authors and do not necessarily represent those of their affiliated organizations, or those of the publisher, the editors and the reviewers. Any product that may be evaluated in this article, or claim that may be made by its manufacturer, is not guaranteed or endorsed by the publisher.
